# A Comprehensive Survey on Deep-Learning-Based Breast Cancer Diagnosis

**DOI:** 10.3390/cancers13236116

**Published:** 2021-12-04

**Authors:** Muhammad Firoz Mridha, Md. Abdul Hamid, Muhammad Mostafa Monowar, Ashfia Jannat Keya, Abu Quwsar Ohi, Md. Rashedul Islam, Jong-Myon Kim

**Affiliations:** 1 Department of Computer Science and Engineering, Bangladesh University of Business and Technology, Dhaka 1216, Bangladesh; firoz@bubt.edu.bd (M.F.M.); ashfia@bubt.edu.bd (A.J.K.); quwsarohi@bubt.edu.bd (A.Q.O.); 2 Department of Information Technology, Faculty of Computing & Information Technology, King Abdulaziz University, Jeddah 21589, Saudi Arabia; mabdulhamid1@kau.edu.sa (M.A.H.); mmonowar@kau.edu.sa (M.M.M.); 3 Department of Computer Science and Engineering, University of Asia Pacific, Dhaka 1216, Bangladesh; rashed.cse@gmail.com; 4 Department of Electrical, Electronics, and Computer Engineering, University of Ulsan, Ulsan 680-749, Korea

**Keywords:** breast cancer diagnosis, neural networks, image pre-processing, imaging modalities

## Abstract

**Simple Summary:**

Breast cancer was diagnosed in 2.3 million women, and around 685,000 deaths from breast cancer were recorded globally in 2020, making it the most common cancer. Early and accurate detection of breast cancer plays a critical role in improving the prognosis and bringing the patient survival rate to 50%. Deep learning-based computer-aided diagnosis (CAD) has achieved remarkable performance in early breast cancer diagnosis. This review focuses on literature considering deep learning architecture for breast cancer diagnosis. Therefore, this study anchors a well systematic and analytical review from six aspects: the model architecture of breast cancer diagnosis, datasets and image pre-processing, the manner of breast-cancer imaging, performance measurements, and research directions.

**Abstract:**

Breast cancer is now the most frequently diagnosed cancer in women, and its percentage is gradually increasing. Optimistically, there is a good chance of recovery from breast cancer if identified and treated at an early stage. Therefore, several researchers have established deep-learning-based automated methods for their efficiency and accuracy in predicting the growth of cancer cells utilizing medical imaging modalities. As of yet, few review studies on breast cancer diagnosis are available that summarize some existing studies. However, these studies were unable to address emerging architectures and modalities in breast cancer diagnosis. This review focuses on the evolving architectures of deep learning for breast cancer detection. In what follows, this survey presents existing deep-learning-based architectures, analyzes the strengths and limitations of the existing studies, examines the used datasets, and reviews image pre-processing techniques. Furthermore, a concrete review of diverse imaging modalities, performance metrics and results, challenges, and research directions for future researchers is presented.

## 1. Introduction

The most commonly occurring cancer in women is breast cancer (BrC). As claimed by the World Health Organization (WHO), BrC was diagnosed in 2.3 million women, and 685,000 deaths were recorded globally in 2020 [1]. In addition, the WHO predicts that the number of new BrC patients will increase by seventy percent (70%) in the next twenty years. Besides, BrC is the 5th-most deadly disease out of distinct cancer types, such as lung, colorectal, liver, and stomach cancers [2]. As per the Global Cancer Statistics 2020 (GLOBOCAN), female breast cancer (FBC) is the most widespread cancer, with new cancer cases of 2.3 million (11.7 percent of total cases) in 2020 [2]. However, male breast cancer (MBC) is a very uncommon cancer. Because of its rarity, treatment is currently mostly based on data derived from FBC treatment, even though MBC has distinct molecular features [3]. FBC is a growing epidemic in South Asian countries because of insufficient knowledge of BrC. Most of the time, it remains hidden, and the majority of patients are diagnosed at the advanced level of the disease in a south Asian country such as Bangladesh [4,5,6]. A cost-effective health service strategy is thus required, which will be affordable to many women in a country like Bangladesh.

The current survival rate of BrC at late stages is low (30%). The early and accurate detection plays a vital role in improving the prognosis and raising the survival rate in patients to 50% [1]. Proper diagnosis of BrC requires correct identification of each stage of cancer, also identifying its category. Different medical images are commonly used for effective BrC diagnosis than any other BrC testing method. Medical imaging modalities such as histopathology (Hp) images, breast X-ray images (mammography), sonograms (ultrasound imaging), and magnetic resonance imaging (MRI) are broadly used for BrC diagnosis [7,8]. A professional pathologist’s experience and subject knowledge are required for a reliable BrC diagnosis. Without these, in most cases, misdiagnosis occurs, especially in the early stages of BrC. However, it is crucial to diagnose BrC at an early stage. In reality, many computer-aided diagnosis (CAD) systems are used to aid physicians in the early diagnosis of breast tumors on mammograms [9]. It is a readily available, fast, reliable, and affordable system for early BrC diagnosis. CAD has been developed during the last decade and has shown advancement in BrC detection accuracy by more than 20% [10]. This system helps physicians and radiologists recognize abnormalities by using different imaging modalities, reducing the rate of mortality from 30% to 70% [11].

Deep learning (DL)-based CAD systems have achieved advancement in the medical field by examining data in radiology [12,13], pathology [14], cardiology [15], pharmacology [16], oncology [17,18], and genomics [19] for diseases’ diagnosis and prognosis. More complex approaches have been used in recent decades for cancer detection based on machine-learning techniques. DL has been widely acknowledged as one of these techniques, demonstrating its efficacy in predicting cancer and its efficacy in prognosis [20]. DL has a higher diagnostic accuracy for detecting breast cancer on mammograms, ultrasound, and DBT in breast-cancer imaging [21]. Currently, clinical treatment of BrC relies upon DL for higher accuracy. In the last few years, many articles have been published on BrC using deep learning [22,23,24]. Deep-learning algorithms can handle the complexities and challenges of automatic diagnosis of BrC with efficiency. So far, many review studies have been done on BrC classification, but few of them are able to provide a clear direction for future researchers. Although these studies have presented a good literature survey of BrC, they could cover a few areas on deep learning. Most of the review studies published on BrC mainly emphasized generic artificial neural networks (ANNs) or traditional ML algorithms [25], where feature extraction is involved for diagnosis. They could not address emerging deep-learning architectures on BrC diagnosis, such as generative adversarial networks (GANs), extreme learning machines (ELMs), etc. Recent studies on BrC introduced several imaging modalities. However, in most cases, previous review studies overlooked emerging imaging modalities, such as infrared thermal imaging, digital breast tomosynthesis, and computed tomography. Although a few review articles are available for digital breast tomosynthesis [26], they could not cover all imaging modalities used in BrC classification. Furthermore, their analysis of deep0learning-based techniques was incomprehensible as they did not give a clear overview of previous research’s strengths and weaknesses. Therefore, this study anchors well a systematic and analytical review of present state-of-the-art deep-learning-based BrC image classification using CAD systems from six aspects: the model architecture of BrC diagnosis, datasets and image pre-processing, the manner of breast-cancer imaging, performance measurements, and research directions to overcome the limitations mentioned above.

**Figure 1:**
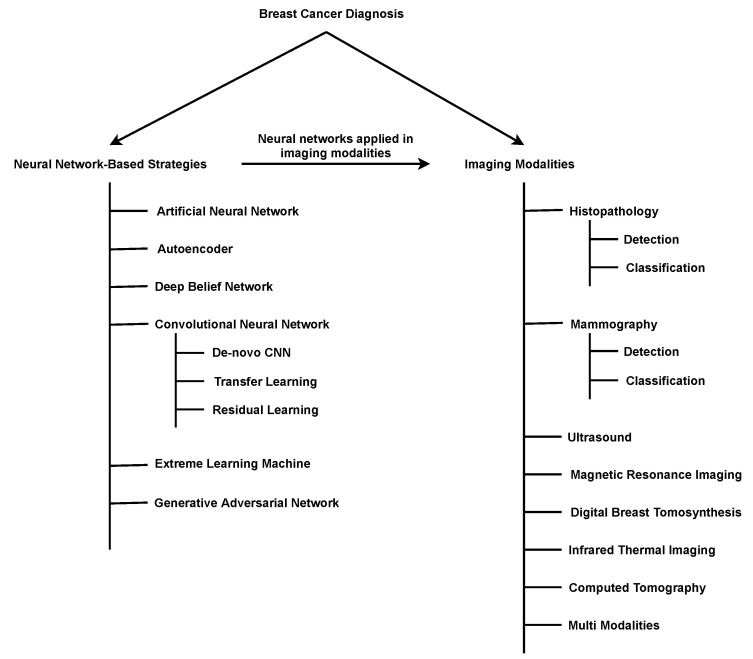
A taxonomy of deep-learning-based breast cancer diagnosis.

Figure 1 illustrates the taxonomy we considered in this review. The BrC diagnosis was divided into six neural network designs that are applied in medical imaging. This medical imaging was divided into eight categories. We have selected studies from 2015 to 2021 based on their popularity to perform this review. In this investigation, we espoused a systematic review methodology that will help future researchers determine the overall skeleton of a deep-learning-based BrC diagnosis. This review gives a clear view of deep-neural-network architectures that were engaged in identifying BrC. This study also addresses the detection and classification of breast cancer utilizing imaging techniques. Finally, this review directs several open research challenges and opportunities for future researchers. We believe that this review serves as a valuable guideline for researchers who want to work on medical image classification pivoting to deep learning-based BrC diagnosis while utilizing diverse types of medical images. Table 1 shows a comparison between the existing surveys and our review.

The rest of the article is structured as follows: We discuss the breast cancer diagnosis methods in Section 2. We provide an overview of the datasets and commonly used image pre-processing methods in Section 3. We present all the categories of imaging modalities in Section 4. We present performance metrics for result analysis of previous studies in Section 5. We also provide challenges and future research directions in Section 6. Finally, Section 7 concludes the article.

## 2. Breast-Cancer-Diagnosis Methods Based on Deep Learning

**Figure 2:**
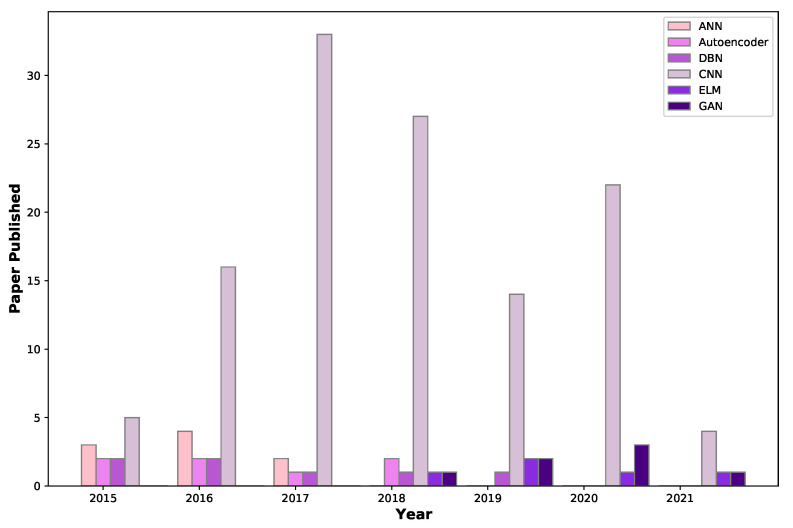
This figure shows the published number of deep-learning-based breast cancer studies in the past 6 years and the current year.

Deep-learning algorithms have recently made significant developments and achieved outstanding performance, inspiring numerous researchers to use deep learning in BrC diagnosis. This deep-learning-based CAD scheme’s benefits include its capacity to identify breast masses as cancerous or normal without lesion segmentation, image feature calculation, and a selection procedure [38]. This section describes the main categories of existing deep-learning-based BrC diagnosis methods, including artificial neural networks, autoencoders, deep belief networks, convolutional neural networks (CNN), extreme learning machines (ELM), and generative adversarial networks (GAN). Figure 2 illustrates the number of studies on BrC diagnosis published each year using these architectures.

### 2.1. Artificial Neural Network (ANN)

**Figure 3:**
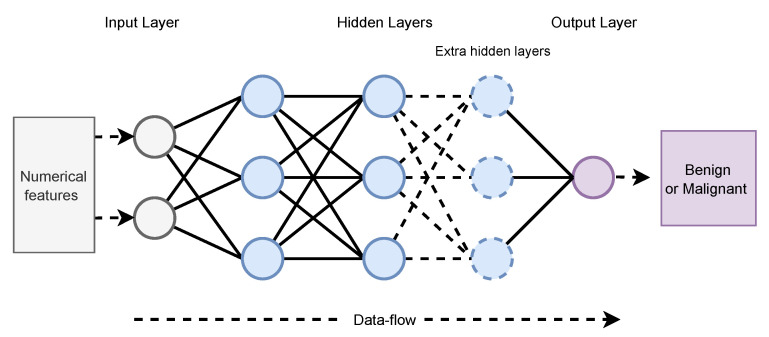
A sample illustration of ANN with multiple hidden layers for breast cancer diagnosis.

An ANN is a mathematical model based on the structure and capabilities of a biological neural network. In terms of computer science, it functions similarly to an actual human brain in terms of receiving, processing, and delivering information. Artificial neural networks (ANNs) play an essential role in the diagnosis of BrC. It is essential to clarify that ANNs do not aim to replace the radiologist but rather help them to ensure their accuracy and reliability. Radiologists who employ ANN will eventually replace those who don’t. Radiologists are being trained on how to spot ANNs’ vulnerabilities and utilize their advantages. Radiologists protect patients from ANNs’ false-positive results [39]. An ANN with multiple hidden layers performs well for complex problems, but it needs more time to train. The basic architecture of an ANN with multiple hidden layers is given in Figure 3. On the other hand, wn ANN with a few layers is simple to create and train and is easy to optimize the training parameters. Furthermore, a small amount of data results in better generalization efficiency. However, it does not perform well on high-dimensional data.

One of the initial works in this area is presented by Abbass [40]. The study applied ANN for BrC detection on the Wisconsin dataset consisting of numerical features. This study demonstrated that the proposed memetic Pareto artificial neural network (MPANN) enhanced generalization and significantly reduced computational costs by comparing their results against an evolutionary programming approach and standard back propagation (BP). However, this work did not consider any feature engineering concept. Another work by Karabatak et al. [41] proposed an approach for automatic diagnosis of BrC with association rules (AR) and a neural network (NN). Here, AR were used to reduce the feature vector’s dimension, and, for classification, a NN was used. The proposed (AR+NN) model provided better performance than the NN model. The suggested method had an accurate classification rate of 95.6% with four inputs and 97.4% with eight inputs. Jafari-Marandi et al. [42] proposed a model named life-sensitive self-organizing error-driven (LS-SOED). It improved the ANN’s performance in decision-making goals. The inclusion of missing values in the WBDC dataset has affected SOED’s best success in terms of accuracy and the misclassification rate. Anton s. Becker [43] proposed a deep-learning-based ANN architecture to detect BrC using the mammography of the BCDR dataset. The diagnostic accuracy results achieved by the proposed ANN architecture were comparable to other recently published deep learning models. However, they did not consider patients with post-surgical changes in their cohort. The BCDR dataset contained only a brief biography of the patient, including patients with minor frightening changes, introducing a bias. Rouhi et al. [44] trained an ANN with limited images to classify benign and malignant cancers in mammograms. Segmentation was done using an ANN and cellular neural networks. A genetic algorithm was also utilized to determine the parameters of the cellular neural network. In Table 2, we summarize the existing studies based on ANN along with their strengths and limitations.

### 2.2. Autoencoder

**Figure 4:**
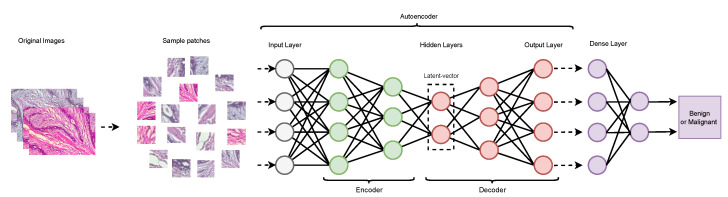
An illustration of the autoencoder model for breast-cancer diagnosis.

An unsupervised learning technique autoencoder applies back-propagation, adjusting the target values equal to the inputs. It is a neural network with three layers: the input layer, the hidden layer, and the decoding layer. An autoencoder converts an input into a hidden layer. Then decoder reconstructs the input from the hidden layer. There are four types of autoencoder: a denoising autoencoder (DAE), a sparse autoencoder (SAE), a variational autoencoder (VAE), and a contractive autoencoder (CAE). The stacked denoising autoencoder (SDAE) is a denoising version of the stacked autoencoder. Auto-encoders give us an advantage by reducing the dimensionality of the data. Autoencoder training entails a large amount of data, processing time, hyperparameter adjustment, and validation of the model. Although an autoencoder is quite similar to principal component analysis (PCA), it is more flexible than PCA. PCA can perform only linear transformation, but an autoencoder can perform both linear and non-linear transformation. The architecture of an autoencoder is shown in Figure 4.

Xu et al. [45] suggested a stacked sparse autoencoder (SSAE) framework that consists of two SAE for the classification of nuclei patches on breast-cancer histopathology images. In an unsupervised manner, the SSAE framework learns high-level features for improved representation of raw input data. The evaluation result from SSAE+ Softmax for nuclei patch classification was better than the conventional softmax classifier, PCA+Softmax, and SAE+Softmax. However, the breast histopathology images were collected from a cohort of only 17 patients. After that, Xu et al. [46] published another study with 537 H&E stained histopathological images. They proposed a method for nuclei detection of BrC by the SSAE framework. However, they did not consider any pre-processing techniques. Another study by Kadam et al. [47] proposed feature ensemble learning with SSAE and proved that it performs better than the SSAE+softmax architecture.

Few studies have utilized autoencoders to reconstruct a dataset and learn more relevant features from gene expression data [48,49]. Feng et al. [50] proposed an SDAE architecture intending to learn significant features from the image patches. It is appropriate for classifying cell nuclei when just a limited number of labeled data are accessible. Cheng et al. [51] utilized SDAE on the two CADx applications to differentiate breast ultrasound lesions and lung CT nodules. The SDAE design includes a noise-tolerance advantage and an automated feature-exploration mechanism. Thus, it is ideal for dealing with the intrinsically noisy quality of medical-image data from diverse imaging modalities. Two traditional CADx algorithms were applied for comparison to demonstrate the outperformance of SDAE-based CADx over the traditional method. Adding additional nodule member slices as training data will offer the SDAE model richer image contexts, enhancing distinction capabilities. Table 3 presents a summary of existing studies based on an autoencoder.

### 2.3. Deep Belief Network (DBN)

**Figure 5:**
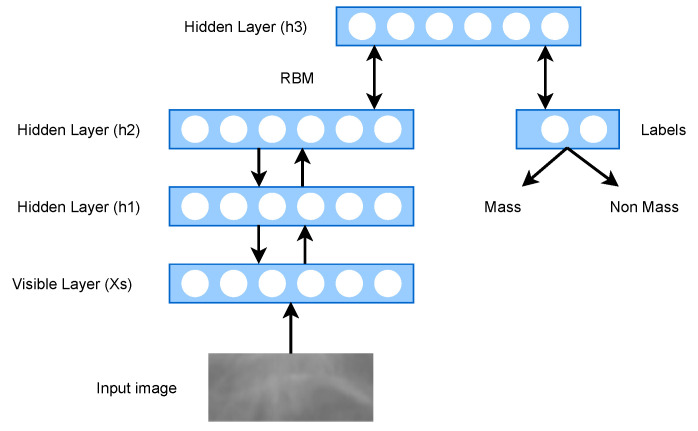
An illustration of the DBN model for breast-cancer diagnosis.

The Deep Belief Network (DBN) is an unsupervised graphical model, which is essentially generative in nature. The DBN is a multilayer belief network, where each layer is a restricted Boltzmann machine (RBM), and they are stacked with each other to form the Deep Belief Network [52]. The initial stage in training DBN is to learn a set of features from visible units using the contrastive divergence (CD) method. Then, the activations of previously trained features are treated as visible units, and, in a second hidden layer, the DBN learns more robust features from the previously acquired visible units [53]. DBN is effective for the following four reasons: DBNs may be fine-tuned like neural networks; they have multiple non-linear hidden layers; they are generatively pre-trained; and they can serve as non-linear dimensionality reduction for input feature vectors [54]. The primary disadvantage is that RBMs are tricky to train. RBM training entails adjusting the parameters so that the model’s probability distribution fits the training data as well as possible. This refers to optimizing the parameters’ likelihood for the training data. Maximum likelihood learning is difficult for undirected probabilistic graphical models because the maximum likelihood parameters cannot be derived analytically. The architecture of DBN is presented in Figure 5.

Abdel-Zaher and Eldeib [54] proposed a CAD scheme for the detection of BrC, and they used DBN’s unsupervised phase followed by a supervised backpropagation neural-network phase (DBN-NN). There were no procedures used for data pre-processing in this work. Another work by Khademi and Nedialkov [55] showed that integrating microarray and clinical data can improve the performance of the model as cancer is a genetic disease. However, due to the curse of dimensionality and small sample size issues, integrating genetic factors in microarray data may result in poor structure and parameter-learning results. The authors solved these issues by using DBN to microarray data.Dhungel et al. [56] suggested a method for breast masses’ segmentation from mammograms. Their proposed model used a structured support vector machine (SSVM) for learning and DBN as a potential function. After that, Dhungel et al. [57] discussed the use of deep CNN and DBN as potential functions in structured prediction models for the segmentation of breast masses. They showed that conditional random field (CRF) and SSVM both can produce the better results than the other state-of-the-art method. Furthermore, they showed that the training and testing time required by the CRF model is less than the SSVM model. Overall, the CRF model has some advantages over the SSVM model. Few studies used DBN for feature extraction and prominent feature selection [58,59]. Zhang et al. [59] suggested a two-layer DL architecture for feature extraction that included the point-wise gated Boltzmann machine (PGBM) and RBM. Although this design performed better, it requires more training time. Table 4 presents a brief overview of existing studies on DBN architecture.

### 2.4. Convolutional Neural Network (CNN)

A convolutional neural network (ConvNet/CNN) is a deep-learning framework that takes an image, assigns weights and biases to distinct features in the image, and distinguishes one image from the other. CNN architecture is built on three main design concepts: local receptive fields, weight sharing, and sub-sampling. The CNN was initially developed to identify two-dimensional image patterns. A CNN is made up of three layers: (1) convolution layers, (2) max-pooling layers, and (3) an output layer.

A detailed overview of the convolutional neural network (CNN) is needed since it is a central tool in BrC classification. CNNs are utilized more often to develop a reliable BrC classification model in previous studies [7,60]. CNNs are utilized with different imaging modalities as they work well with images. However, a large number of images are needed to train a CNN. It is difficult to achieve good performance with a limited number of images. Moreover, it is difficult to obtain adequate training data because obtaining labeled datasets in medical imaging is costly. However, CNN has many advantages. Opposed to other classification methods, a ConvNet requires much less pre-processing. Feature extraction and classification are entirely combined into a single CNN architecture. Lastly, it is resistant to picture noise and local geometric distortions. Therefore, studies used CNNs to extract useful features from medical images and to perform BrC classification with them [61]. De-novo CNNs (CNNs trained from scratch) and TL-based CNNs (pre-trained CNNs) are mainly used in BrC classification [62,63,64].

#### 2.4.1. De-Novo CNN

**Figure 6:**
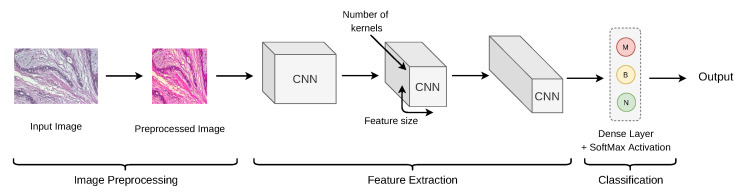
An illustration of CNN-based model for breast cancer diagnosis.

Several studies used a CNN with few layers or multiple layers by training it from scratch. We refer to them as De-novo CNN. It is possible to achieve better image classification results using deep convolutional neural networks (DCNNs) with several layers and a large quantity of data. The lightweight CNNs trained from scratch outperform typical ImageNet-transferred models (pre-trained models). A study by Arevalo et al. [7] proved that the smaller CNN built from scratch performed better than the pre-trained model. A basic workflow with CNN in breast cancer diagnosis is given in Figure 6.

De-novo models are further divided into two types in BrC classification: uni-dataset model (UDM) and multi-dataset models or cross-origin models (COM). Existing research utilized benchmark datasets with CNNs for image classification, but a few studies used their own dataset, making the comparison difficult for other researchers [65,66,67,68]. CNNs are also used for feature extraction and image segmentation [69,70]. Araújo et al. [71] experimented with the extracted features of CNN to train an SVM classifier and showed that a CNN’s ability to extract significant features is excellent. Spanhol et al. [60] applied a CNN using fusion rules, such as Max, Product, and Sum, for histopathological image classification, and it led to an improvement of about 6% accuracy when compared to other methods utilizing the same dataset. They have proposed a strategy based on patch extraction from images, which helps to deal with high-resolution images with the same CNN architecture designed for low-resolution images. Albarqouni et al. [72] proposed a novel concept of collecting training data using crowdsourcing for mitosis detection. Their proposed multi-scale CNN (AggNet) model can handle data aggregation through the learning process with an additional crowdsourcing layer. They also validated their model on a benchmark dataset. Further, in Table 5, we present a brief overview of existing studies on De-novo CNN.

#### 2.4.2. Transfer Learning (TL)-Based CNN

Transfer learning is an efficient approach for dealing with small datasets by allowing pre-trained networks to be fine-tuned and adjusted to solve problems from a particular domain or imaging modality. The weights of the model are pre-initialized when utilizing a pre-trained version, as opposed to being randomly initialized while training from scratch [63]. However, TL might be tricky because it can easily overfit. Huynh et al. [63] claimed to be the first study to apply a pre-trained CNN (AlexNet) as a fixed feature extractor for diagnosing medical images, and the classifier performed better on features extracted with a pre-trained CNN. AlexNet, VGG-Net, GoogLeNet, and ResNet are widely used as TL-based models. The pre-trained models are trained on natural images and often have a deep architecture to learn a large number of class labels; for example, AlexNet was trained for 1000 class labels and had five convolution layers and three fully connected layers. A study by Samala et al. [73] suggested a multi-task transfer learning DCNN for the classification of breast masses. This study showed that multi-task DCNN could still be effective when the number of images is limited. A study by Mendel et al. [74] experimented with a pre-trained CNN and extracted useful features from limited data (78 images). On four medical imaging applications, Tajbakhsh et al. [75] compared de-novo and fine-tuning models. The authors proved that fine-tuned (TL-based) CNNs surpassed CNNs learned from scratch (de-novo) and were more resistant to the training set scale.

Moreover, it is also showed that TL from different domains (natural images) has no significant effect on the performance of medical imaging tasks. Transfer learning provides minimal performance benefits [76]. Researchers used two methods to use TL for BrC classification. First, they only fine-tuned the last layer of the model; we refer to it as fine-tuned model in the last layer (FTM-LL) [22]. Second, one or multiple layers of the pre-trained network are replaced with newly generated layers before retraining the network with a training sample, referred to here as the fine-tune model with modified layers (FTM-ML) [77,78]. Table 6 illustrates a brief overview of existing studies on a TL-based CNN.

#### 2.4.3. Residual Learning (RL)-Based CNN

Recently few studies utilized residual learning for BrC diagnosis [79,80,81]. The main components of residual networks are residual blocks. It uses skip connections to leap across the layer in order to keep the network from experiencing the problem of a vanishing gradient. When compared to stacked CNN, the addition of residual blocks in the network increases representation power, leads to faster convergence, and lowers training errors [82]. A work by Singh et al. [83] proposed a pre-trained model with FFDM images, which was utilized for DBT images. This study used two fine-tuning methods: (1) fine-tuning the last two layers and (2) fine-tuning only the optimal layers. The authors compared two fine-tuning approaches, and the findings revealed that fine-tuning the last two layers produced the best AUC (AUC = 0.847).Toğaçar et al. [79] proposed a novel approach with a residual architecture built on attention modules called the BreastNet model. Each image datum of the BeakHis dataset was augmented before feeding the data to the model. The model then selected and processed vital image regions using attention modules for each image. Another study by Gour et al. [80] proposed a residual learning-based approach with 152 layers of a CNN named RestHis. The model can learn discriminative and rich features and classify histology images into benign and malignant types. They designed a data-augmentation technique and achieved better performance. More notably, the ResHist model beats pre-trained networks such as AlexNet, GoogleNet, VGG16, VGG19, Inception-v3, ResNet50, and ResNet152. ResHist categorizes the whole slide image, preserving the global information of histopathological images. However, it consumes a significant amount of processing power and takes time to train. When only raw images in BreakHis were trained and evaluated, the classification accuracy attained with their model was reported as 82.12%, 82.98%, 80.85%, and 81.83% for 40×, 100×, 200×, and 400× magnification images, respectively. Hu et al. [81] proposed a CNN-based network named myResNet-34 with a deep residual learning structure. Accuracy rates with myResNet-34 on the raw images of BreakHis were 86.90%, 84.62%, 85.34%, and 81.06%, for 40×, 100×, 200×, and 400× magnification images, respectively. Such outcomes proved the effectiveness of their model compared to ResHist [80]. With their proposed augmented strategy, classification accuracy on 40× images obtained an accuracy of 93.63%, which was a 6.73% improvement comparing to the result without data augmentation. Li et al. [84] experimented with residual learning for breast-density classification using mammograms from two datasets. The authors combined deep residual networks with integrated dilated convolutions and attention methods to improve the networks’ classification performance. The proposed method performed better on their own dataset than the publicly accessible dataset. Table 7 illustrates a brief overview of the existing studies on RL-based CNN.

### 2.5. Extreme Learning Machine (ELM)

**Figure 7:**
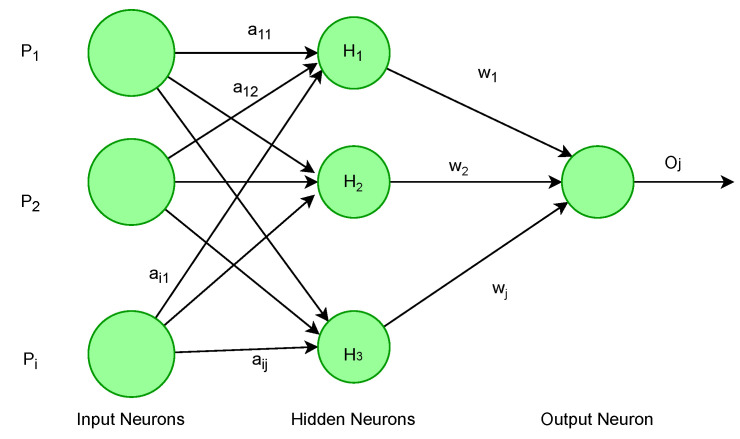
A basic extreme learning machine architecture.

The extreme learning machine (ELM) is an ANN variant with a high potential for handling BrC classification. ELM is a feed-forward neural network often used for clustering, pattern recognition, classification, and regression with a single layer or hidden layers. This algorithm is based on random initialization of input weights and biases and the analytic calculation of the output weights. Therefore, the ELM is insensitive to manual parameters setup. ELMs have become renowned for tackling many complex issues because of their benefits of high learning speed and low computing cost [85,86]. ELMs are not as accurate as standard neural networks, but they can be helpful in dealing with situations that need real-time network retraining. Moreover, the training process of the ELM is very time-consuming. The architecture of the ELM is given in Figure 7.

A study by NEMISSI et al. [87] used an ELM with multiple activation functions for the hidden neurons and optimized them using a genetic algorithm. The proposed method improved generalization performance. However, the model assessment was inadequate as they only used accuracy for model assessment. Based on CNN’s feature extraction and representation capabilities and ELM’s classification resilience, an interactive cross-task extreme learning machine (ICELM) was suggested [88]. First, high-level features are retrieved utilizing deep transfer learning and double-step deep transfer learning. The high-level feature sets are then used as regularization terms to boost the performance of the classification. Wang et al. [89] proposed a method for mass detection. A CNN was utilized to extract features and unsupervised (US-ELM) clustering for clustering sub-regional features. Then, an ELM classifier was used to feed the fusion of features (features extracted with CNN, density features, morphological features, and texture features). ELM classification with fusion deep feature sets achieved higher performance in BrC detection. Another study by Ronoud and Asadi [90] employed a combination of ELM, DBN, and backpropagation (BP) to intelligently pick the weights among the final hidden layer and the output layer rather than randomly. Using non-random and more appropriate network weights at the beginning of the algorithm helps converge earlier and leads to greater classification results. DBN can only be pre-trained until the last hidden layer, but the weights are selected randomly among the output layer and the previous hidden layer. Another study by Muduli et al. [91] performed feature reduction and classification by fusing the extreme learning machine and the moth flame optimization technique (MFO-ELM) for BrC classification. The feature reduction may lead to data loss. Table 8 depicts a brief overview of the existing studies on ELM architecture.

### 2.6. Generative Adversarial Network (GAN)

**Figure 8:**
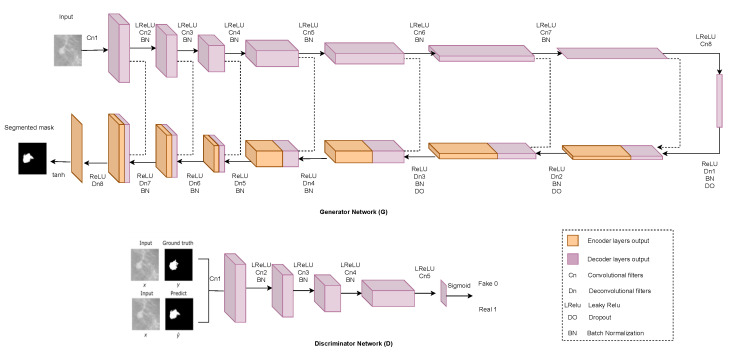
A cGAN Architecture: generator G (**top**) and discriminator D (**bottom**).

Generative adversarial networks (GANs) are deep-learning-based generative models. The GAN model framework consists of two sub-models: a generator model for creating new instances and a discriminator model for determining whether the produced examples by the generator model are genuine or fake. The generator model produces new images from the features learned in the training data that resemble the original image. The discriminator model predicts whether the generated image is fake or real. Yann LeCun, Facebook’s AI research director, has labeled GANs “the most interesting topic in ML in the last ten years [92].” The drawbacks of not having enough pictures to train a classifier are well known. For some types of medical imaging data, such as infrared thermal imaging, it is difficult to collect a large-enough number of images to train a classifier. Obtaining labeled data is a time-consuming manual process. As GANs do not require labeled data, they are particularly valuable for BrC classification. However, the major disadvantage of using a GAN is the lack of intrinsic evaluation metrics. The model can be unstable during training and can take a considerable amount of time. GAN’s other weaknesses includes its inability to control what images GAN will produce and the lack of control over the style of generated images. This is where conditional GANs (cGAN) and style GANs come into play since they let us direct the generator as to which image to generate, and control over the style of produced images at various levels of detail is introduced. Another type of GAN that is based on the cGAN is Pix2pix. Pix2Pix GAN is a generic method for translating images. A GAN architecture is given in Figure 8.

Shams et al. [93] constructed a deep generative multi-task (DiaGRAM), which was built upon the ensemble of CNN and GAN to reduce the mortality rate in BrC. DiaGRAM incorporated strategies for achieving extremely accurate mammography diagnoses. It synthesized a GAN with a deep classifier to learn features. Another study by Singh et al. [94] used cGAN for breast-tumor segmentation inside a mammogram’s region of interest (ROI). The generative model learns to detect the tumor region and generates a binary mask that defines it. As a result, the adversarial network learns to differentiate between actual (ground truth) and synthesized segmentations. It drives the generative network to generate binary masks that are as realistic as possible. Then, using a CNN-based shape discriminator, they classify the binary masks.

Moreover, GANs have recently been used as image-augmentation techniques to solve the drawback of insufficient training data. Thuy and Hoang [95] created the fake BreakHis datasets from the original BreakHis dataset using StyleGAN and conditional GAN (Pix2Pix). Guan and Loew [96] used GAN as a new mammographic image generator from the DDSM database and used CNN as the discriminator of GAN. GAN performed about 3.6% better than other image-augmentation techniques. Fan et al. [97] planned to produce super-resolution apparent-diffusion-coefficient (SR-ADC) images with an SR generative adversarial (SRGAN) and an enhanced deep SR (EDSR) network, as well as bicubic interpolation. Another work by Swiecicki et al. [98] used digital breast tomosynthesis data for detecting anomalies by using GAN to complete an image. The detection system reported in this study yielded promising findings, as it was able to identify suspicious spots without the need for training images with abnormalities. Tien et al. [99] proposed Cycle-Deblur GAN combining CycleGAN and Deblur-GAN to improve the image quality of breast-cancer patients’ chest CT images. Table 9 illustrates a brief overview of the existing studies based on GAN architecture.

## 3. Datasets and Image Pre-Processing

A benchmark dataset and data pre-processing techniques are crucial for BrC classification. This section provides an in-depth review of public databases used in different research for BrC classification in Section 3.1. Furthermore, the standard pre-processing methods used in previous studies are listed in Section 3.2.

### 3.1. Dataset

Several datasets have been published for BrC diagnosis. A number of studies used medical-image datasets, whereas clinical data were utilized in few studies. The most popular and widely used dataset of clinical information is the Wisconsin Breast Cancer Dataset. Dr. William H. Wolberg created the Wisconsin Breast Cancer Database (WBCD) at the University of Wisconsin Hospitals [100]. This dataset contains 699 cases and 11 attributes for each case. The independent attributes are clump thickness, uniformity of cell size, uniformity of cell shape, marginal adhesion, single epithelial cell size, bare nuclei, bland chromatin, normal nucleoli, and mitoses. Deep-learning algorithms need a massive dataset for training. The lack of data is a major obstacle in applying deep-learning algorithms to medical diagnosis. Many researchers used their personally collected data in BrC classification; we referred to these datasets as private datasets (PD). The most serious critique raised in our study is that researchers use such a small dataset to train and validate the suggested methodologies. Most researchers used transfer learning to tackle this issue. However, several studies used publicly accessible datasets. Histopathological images provide significant information and are thoroughly studied by specialists to determine the patient’s present condition. Histopathological images can be found in the BreaKHis, BICBH, ICPR 2014, and TUPAC16 databases.MIAS, mini-MIAS, DDSM, and CBIS-DDSM are popular datasets of mammograms.

The Digital Database for Screening Mammography (DDSM) is the most-often-used database. It is the largest public database, with 2620 instances containing two images from each breast, namely, the mediolateral oblique (MLO) and craniocaudal (CC) view, with a total of 10,480 images containing all forms of findings ranging from regular images to images containing benign and malignant lesions. However, it is no longer supported. Clark et al. [101] recently published CBIS-DDSM, an improved and simplified variant of the DDSM dataset for the assessment of CAD processes in mammography. It comes with a readily usable dataset as well as enhanced ROI-segmented images. The dataset includes 753 microcalcification cases and 891 mass cases, respectively. While being the oldest available database, the Mammographic Image Analysis Society Digital Mammogram Database (MIAS) [102] is still widely used in research. The MIAS database is made up of 161 cases and 322 digitized MLO images with various findings, including benign and malignant lesions and regular images. This database’s resolution has been diminished to construct a new database called mini-MIAS [103]. Although images are still available, it is currently not supported. The BancoWeb LAPIMO dataset is accessible to users after registration. It includes 320 cases and 1473 images with MLO, CC, and magnification views and images with benign and malignant findings. The INbreast dataset has mammogram images from screening, diagnostic, and follow-up cases. The carefully associated ground truth annotation by a specialist is the work’s most distinguishing feature. Ultrasound, CT scans, and DBT datasets are usually private, though we found a publicly available CT scan dataset called LIDC [104] and an ultrasound dataset called BCDR [105]. Thermograms are often kept in private image databases that are only accessible to internal diagnostic applications, and, most of the time, only the patient and his physician have access to the images [106]. Datasets can differ based on their imaging modality, class, and image format. The available datasets have distinct image formats. A few datasets contain images in DICOM format, some in TIFF format, and others in PNG format. Therefore, we categorized these datasets based on these categories with their accessible link in Table 10. Maintaining a publicly available database is crucial for expanding the possibilities for study in the BrC classification field.

### 3.2. Image Pre-Processing

This section discusses various pre-processing techniques used for medical-image processing in BrC diagnosis. We concentrated on image pre-processing since many studiesutilized imaging modalities, and just a few studies used patient numerical data. Generally, for BrC, image pre-processing tasks include augmentation, ROI extraction, scaling, image normalization, and enhancement to remove artifacts and features. The application of raw images (without pre-processing) in machine learning usually deflects the results of classification and may sometimes lead to poor results as output.

In Table 11, we provide the pre-processing techniques, along with their advantages, that were performed in BrC classification. The table shows that most studies (45 out of 80) employed image augmentation as a pre-processing task. By using image augmentation, researchers expand the number of images synthetically. Image augmentation is used because large numbers of annotated medical images are not available. Moreover, we found that 30 out of 80 studies extracted the region of interest (ROI) from BrC images. Instead of utilizing the full image, which generally contains unnecessary information, neural networks (NNs) can learn representations associated with normal and abnormal areas. In addition, only a few studies (20 out of 80) decreased the size of images before feeding them into DNNs. When images are directly given into DNNs, rescaling is a necessary process, such as CNNs’. However, fewer studies (25 out of 80) employed image-normalization and -enhancement methods prior to BrC categorization. This procedure reduces the noises that are high or low intensity, makes all images uniform, and helps DNNs produce accurate features for normal and abnormal parts of BrC tissue. However, few studies (6 out of 80) remove various artifacts such as labels, opacity, wedges, markers, and pectoral muscles from images. Before conducting a BrC diagnosis, this method removes non-breast areas from the image. Few studies execute artifact removal from images because it is only needed in specific image types such as mammograms, ultrasound, and MRI. Finally, stain normalization pre-processing techniques were applied in some studies (12 out of 80). A stain normalization method aids in the reduction in inconsistencies seen in histological BrC images. Sert et al. [115] showed that pre-processing poses an enormous effect on the classification performance.

## 4. Imaging Modalities

Imaging is crucial for the diagnosis and treatment of BrC. Therefore, this research demonstrates that BrC diagnosis depends on nine distinct categories of medical imaging modalities. The nine medical imaging modalities are histopathology, mammography, ultrasound, magnetic resonance imaging, digital breast tomosynthesis, infrared thermal imaging, computed tomography, and mass-spectrometry imaging, and their combination is referred to as multi-modalities.Two-view mammography and histopathology are the most basic and widely used imaging modalities in breast imaging. According to Table 5 and Table 6, the majority of the work was done in either histopathological images (Hps) or breast mammograms (Mgs), also known as X-ray grayscale images. The accessibility of images may be the primary explanation for the vast number of publications that use Mg and Hp images. Researchers typically categorize BrC into two primary cancer forms, benign and malignant subtypes. The number of studies released for ultrasound images is the third-largest [24,155]. Another imaging method called breast magnetic resonance imaging (MRI) is a costly procedure with minimal availability. These must be the cause of the lower adaptivity of MRI in research as very few studies utilized this imaging method [156,157]. Digital breast tomosynthesis and computed tomography imaging methods were used only in a few studies [67,124]. The infrared thermal imaging method has gained popularity recently in BrC classification. Only a few studies used this imaging method because the dataset of thermograms is not publicly available [148,158]. Unfortunately, none of the researchers utilized positron-emission tomography (PET) and scintimammography imaging techniques. We describe popular imaging methods in more detail in the following sections. We sequentially placed the imaging methods based on their popularity.

### 4.1. Histopathology (Hp)

**Figure 9:**
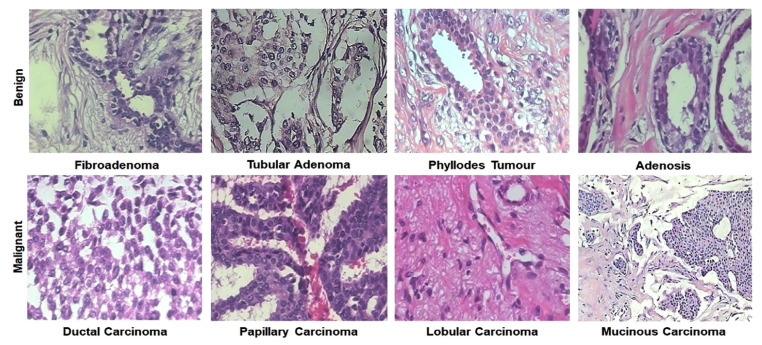
Representative H&E stained images from the BreakHis dataset.

Histopathology refers to the microscopic examination of tissue. It can be said that histopathology is known as the examination of a biopsy or tissue by a pathologist. In BrC detection and diagnosis, the histopathology image dataset is very popular. Histology images from the BreakHis dataset are given in Figure 9. For BrC diagnosis from the histopathological image, at present, the deep-learning approach is the best method because of their high accuracy and efficiency [159]. We obtained some popular histopathology image dataset sites, including BreaKHis, ICPR2014, TUPAC16, etc. This section describes and categorizes some deep-learning-based models for diagnosing BrC histopathology images, which are discussed below:

#### 4.1.1. Detection

Breast cancer is diagnosed by histological examination of hematoxylin and eosin (H&E)-stained tissue sections by expert pathologists. It is a tedious and time-consuming process [78]. Detecting mitotic tumor cells per tissue area is one of the most primary indicators of BrC prognosis. Usually, pathologists count it manually, but automating the process could reduce its time and costs, minimize errors, and improve the accuracy of the results. As a result, researchers attempted to automate the process using deep-learning networks.

Cireşan et al. [160] suggested a method for mitosis detection with histology images using a deep max-pooling convolutional neural network in 2013. They used the MITOS dataset. This method outperformed all other related competitors on the first public annotated dataset of BrC histology images with a 0.78 F1-score. However, their model was smart enough to detect mitosis but has some drawbacks. Firstly, it takes more time to scan pixel by pixel and makes it impractical for clinical practice. Secondly, it needs vast numbers of training samples, which may cause over-fitting during the training process. To overcome these shortcomings of previous methods, Chen et al. [161] presented a novel deep cascaded neural network model (CasNN). The CasNN can detect mitosis approximately 60 times faster than the previous model suggested by Cireşan et al. [160]. MonjoySaha et al. [162] proposed a deep-learning model using the CAFFE deep-learning framework. This model comprises deep architecture and handcrafted features. The deep architecture consists of five convolution layers, four max-pooling layers, four ReLU, and two fully connected layers. As a compendium, we can say that this model outperformed Cireşan et al. [160] with a 0.90 F1-score, though it has some shortcomings like a restricted magnification size and an increased false-positive rate, and it can miss important mitotic figures. Proper concentration and expertise in image patching can subdue those limitations to a greater extent. Balkenhol et al. [163] explored fully automatic mitotic counting by a CNN using two cohorts. The authors proved that the evaluation modality (glass slides, whole slide image (WSI)) had no effect on manual mitotic counting. However, their study was constrained by the tumor selection process in cohort A, which was based on the first mitotic count of one of the included observers during regular clinical workup.

#### 4.1.2. Classification

Pathologists examine the histological characteristics of hematoxylin-and-eosin-stained tissue sections for signs of cancer and abnormalities in typical structures of the breast parenchyma throughout the examination. It is worth noting that early categorization of breast samples as benign or malignant is crucial for selecting the best potential cure. Thus, CAD systems examine histopathological images of sample tissue, identify histological patterns that are linked to cancerous and non-cancerous states, and classify histopathological images as benign or malignant [80]. The standard procedure of histopathological image classification splits a large image into smaller patches, which are then classified using a classifier. However, this technique confronts two challenges. First, the high-resolution features of histopathology images are not completely used to increase classification accuracy. In addition, the pathological image patch’s feature representation is not rich.

Spanhol et al. [60] suggested a method for BrC histopathology image classification using a CNN in 2016. They used an existing CNN, and, for training the CNN architecture, they proposed several strategies that allow dealing with high-resolution images as input to an existing CNN without changing the CNN architecture designed for low-resolution images. This method shows higher accuracy compared to the other traditional machine-learning methods trained on the same dataset. Yan et al. [68] presented a method for extracting richer multilevel features and combined the benefits of CNN and RNN, preserving both short-term and long-term spatial correlations between patches. They started by dividing the high-resolution pathology images into tiny patches. The CNN is then utilized to extract the patch’s richer multilevel features, and the RNN is used to classify those images. However, to accurately classify benign images, they need more diverse data. Another study by Wang et al. [69] proposed a novel network (FE-BkCapsNet) for automatic classification of BrC histopathological images by fusing convolution features and capsule features, which is beneficial for clinical diagnosis.

### 4.2. Mammography (Mg)

**Figure 10:**
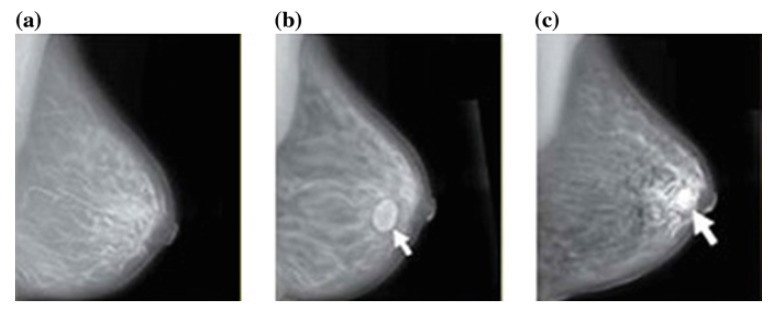
Breast cancer mammogram images from the DDSM dataset: (**a**) normal, (**b**) benign (not cancer), and (**c**) cancer.

Mammography is the second most frequently and extensively used technique for diagnosing BrC. This is because it provides manual nature, variability in mass appearance and a low signal-to-noise ratio. However, mammography misses or misdiagnoses constitute a significant percentage of breast masses. Therefore, it has the potential to produce a high rate of false positives, which may or may not turn out to be cancerous. Thus, mammography screening programs have been criticized for their high recall rate, which results in needless biopsies. Although the increased use of innovative adjunctive techniques such as tomosynthesis may reduce recall rates, we obtained some popular mammography image dataset sites, including MIAS, mini-MIAS, DDSM, CBIS-DDSM, INbreast, BCDR, TCGA, etc. Mammograms from the DDSM dataset are given in Figure 10. Sadeghi et al. [143] proposed an approach that was applied to 170 images of the Mammographic Image Analysis Society Mini Mammographic database. The lack of data on the use of digital mammography is a major drawback to research in this domain [164].

#### 4.2.1. Detection

Mass detection using mammography can save a patient’s life suffering from breast cancer. Kooi et al. [66] proposed a deep-learning model where a huge dataset of approximately 45,000 images were used in a CNN-trained system. This CNN-trained system is unbeaten compared to other state-of-the-art methods in the mammography CAD systems. They have compared the CNN model to a group of three expert readers on a patch level and have proved that the human readers and this CNN model have similar performance. Suzuki et al. [165] advents a method of mass detection by deep CNN for mammographic computer-aided d00000iagnosis. The authors adopted a DCNN architecture consisting of eight layers with weight, including five convolutional layers and three fully-connected layers that can overcome the shortcomings of a conventional CNN that requires a large number of training data. Their proposed model, DCNN with transfer learning, is also applicable where data are limited.

Akselrod-Ballin et al. [166] found a method to detect any abnormalities in mammograms. While the majority of prior research in mammography has focused on abnormality categorization rather than detection and localization, they initialized a fundamental deep-learning method for detecting masses and calcifications. Compared with the current state-of-the-art techniques, this method runs significantly faster. Benzebouchi et al. [138] proposed a CNN strategy for BrC detection utilizing segmented data from DDSM. They suggested an architecture that avoids the fetch of the traditionally handcrafted feature phase by performing feature extraction and classification at the same time inside the same network of neurons. The suggested approach has higher classification rates, allowing for a more-accurate diagnosis of BrC. Akselrod-Ballin et al. [167] combined machine-learning and deep-learning approaches to detect BrC early and applied it to digital mammography images and electronic health records. The model was trained using 9611 mammograms. The algorithm achieved a region under the receiver operating characteristic curve (AUC) of 0.91 for the malignancy prediction goal. Despite achieving a significant outcome, the model cannot distinguish between calcifications or mass.

#### 4.2.2. Classification

The automatic classification of lesions in medical images is essential for a variety of clinical applications. Arevalo et al. [7] suggested a method for mammographic mass lesion classification with CNN in 2015. The proposed method uses CNN to learn mammography mass lesions and then feeds them to an SVM classification stage. They used the BCDR-F03 dataset, and it is a supervised learning approach. The performance result was 86% in terms of the area under the ROC curve (AUC). After that, Arevalo et al. [168] proposed another method to automatically acquire discriminative features using deep-learning methods while avoiding the creation of specialized hand-crafted feature detectors. The authors showed that the performance was improved after combining learned features and hand-crafted features compared with the previous method. Chakravarthy and Rajaguru [169] shed new light on determining whether the input mammograms are normal or abnormal. From three datasets (CBIS-DDSM, MIAS, and INBreast), they collected mammograms and utilized deep extracted features from ResNet-18 with their suggested Improved Crowd-Search Optimized Extreme Learning Machine (ICS-ELM) algorithm. The suggested approach achieved the highest overall classification accuracy, with 98.266% for the INbreast, 97.193% for the DDSM, and 98.137% for the MIAS datasets.

Few studies performed both detection and classification of cancerous tumors [170,171]. A study by Ribli et al. [172] proposed a faster R-CNN model to detect and classify malignant or benign lesions on mammograms, and the model uses mammograms as the only input without the need for human involvement. This approach achieved 2nd place in the Digital Mammography DREAM Challenge with AUC = 0.85. When employed as a detector, the system achieved great sensitivity with relatively few false-positive marks per image on the publicly available INBreast dataset. The use of a limited size of the publicly accessible pixel-level annotated dataset is a drawback of this procedure. While this method’s classification accuracy wastested on a large screening dataset, its detection performance can only be evaluated on the tiny INBreast dataset. Al-Masni et al. [171] proposed YOLO-based CAD systems that also detect and classify BrC masses. It is capable of handling the most difficult cases of various breast abnormalities. The proposed approach uses an ROI-based CNN called You Only Look Once (YOLO). The proposed CAD system consists of four major stages: mammography pre-processing, feature extraction using deep CNN, mass detection with high confidence, and using fully connected neural networks (FC-NNs) to classify mass. The suggested CAD system identified the mass location with an overall accuracy of 99.7%, as demonstrated by five-fold cross-validation testing. The suggested method also identifies between benign and malignant tumors with a 97% overall accuracy. The YOLO-based CAD method can identify masses over the pectoral muscle or dense area, which are regarded as the most-difficult cases of BrC. Dembrower et al. [170] compared the DL-based risk method and density-based methods and found that the DL-based model was better. The density-based models performed worse for more-aggressive cancers, but the DL-based risk model did not. One of the limitations of this study is that the validity was based on a temporal approach instead of external validation. Another drawback is that even though they did not want to detect current cancer, early symptoms of the tumor may have affected the risk scores.

### 4.3. Ultrasound (Us)

Ultrasound imaging is considered a vital step in detecting breast lesions in computer-aided diagnosis systems. Breast elastography is the latest sonographic procedure that offers additional characterization detail on breast lesions in addition to traditional sonography and mammography. Similar to a clinical palpation test, this procedure clarifies the strain or stiffness of a lesion. Yap et al. [8] proposed the exploitation of deep-learning approaches to detect breast ultrasound lesions and analyzed three distinct methods: a patch-based LeNet, a U-Net, and a transfer learning approach using a pre-trained FCN-AlexNet. This study compared and contrasted two conventional ultrasound image datasets obtained from two separate ultrasound systems. Dataset A contains 306 images (60 malignant and 246 benign), whereas Dataset B has 163 images (53 malignant and 110 benign). The deep-learning techniques exhibited an overall improvement in the true-positive ratio, the false-positive ratio, and the F-measure when predicting both datasets. Cao et al. [173] evaluated the performance of several up-to-date object-detection strategies for breast-tumor detection. To gain that, they accumulated a new dataset containing 579 benign and 464 malignant lesion samples and the associated ultrasound images manually annotated by professional physicians. From the total experimental findings, it is clear that the proposed model outperforms other models in terms of both accuracy and recall. At this time, they only detected the tumor regions by using bounding boxes. However, they plan to further investigate the automatic segmentation of tumor areas in the future.

Wang et al. [174] proposed a fundamental 3D convolutional network for automatic detection of cancer. Their contribution is twofold. At first, the authors proposed an aperture loss function to give a voxel-level adaptive threshold to distinguish cancer and non-cancer, gaining more sensitivity with low FPs. Secondly, they proposed a densely deep supervision (DDS) mechanism to enhance the sensitivity effectively by using multi-scale discriminative features of all layers. Liu et al. [175] adopted a CNN to categorize ultrasound images and assess the tumor threatening. To enhance BrC diagnosis performance with CNN, they synthesized domain knowledge and regulated multi-task learning in the training process. Separating images with lump and normal findings yielded the desired results. Furthermore, despite the fact that this is not a segmentation task, the trained classifier’s activation map can properly concentrate the mass areas in images. In recent years, automatic whole breast ultrasound (ABUS) has drawn attention to BrC identification and diagnosis applications. A 3D multi-view tumor detection system for ABUS volumes was introduced in this article [155]. According to experimental findings, their method achieved a sensitivity of 95.06% with 0.57 false positives (FPs) per volume. The proposed approach is more efficient and generic than current detection methods. However, checking ABUS volumes is a time-consuming process that can skip any subtle tumors.

### 4.4. Magnetic Resonance Imaging (MRI)

Magnetic resonance imaging (MRI) is a noninvasive test that creates representations of the body’s interior, such as the breasts, lungs, liver, and bones, using magnets and radio waves. An MRI does not use radiation and is thus considered a better examination. MRIs produce more-detailed images [176]. As a result, breast MRI images provide additional detailed views of soft breast tissues than Mgs, Us, or CT images. When cancer has been diagnosed, the doctor usually demands an MRI to gain detailed knowledge on the disease’s extent. However, MRI can miss any cancers that a mammogram may diagnose. As a result, MRI is often recommended in addition to a mammogram test. An MRI scan, unlike CT scans and X-rays, does not use potentially dangerous ionizing radiation. Breast magnetic resonance imaging (MRI) is well-known for its high sensitivity and specificity. However, MRI has only been used in a few studies to classify BrC as MRI datasets are not publicly available [177,178,179,180].

Bevilacqua et al. [116] proposed a CAD system to classify breast lesions from MR images to support radiologists. They were able to obtain the excellent result by designing a GA-optimized ANN, which resulted in an 89.77% average accuracy and a 100% best accuracy. Another study was carried out to assess the feasibility and function of a novel deep-learning technique using multiparametric breast magnetic resonance imaging (mpMRI), with MRI scans performed on a 3T magnet. They showed that the integrated MPDL system correctly segmented and identified various breast tissues from multiparametric breast MRI [156]. However, in practice, there are certain technological constraints when using the MPDL network. The feasibility of high-resolution dynamic contrast-enhanced magnetic resonance imaging (DCE-MRI) at 7T had not been investigated until a study was conducted in this context. The goal of this feasibility study was to see whether the PKs of ultra-high-field DCE-MRI of the breast at 7T could distinguish benign and malignant breast tumors [157]. Unfortunately, ultra-high-field DCE-MRI utilizing PK analysis of malignant breast tumors was unable to distinguish between different molecular subtypes, the histologic tumor grade, or the BrC proliferation rate. Due to the exploratory aspect of ultra-high-field DCE-MRI at 7T with PK analysis, the sample size was limited.

### 4.5. Digital Breast Tomosynthesis (DBT)

Each image in traditional two-dimensional mammography depicts a superimposition of breast characteristics, leading to composite densities (superimposition of normal glandular tissue from several breast regions to simulate a mass) and overlying normal glandular tissue pathology masking. DBT was created to address these issues [181]. DBT machines come in a variety of configurations, but the basic idea is that they take multiple images from different perspectives of the compressed breast and then reconstruct the images to generate a pile of “slices” that can be browsed over at a workstation. This enables radiologists to “unpick” composite densities and unmask and analyze the mammographic morphology of various pathologies. Potential drawbacks of the method include greater patient radiation exposure, more time for radiologists to read the images (as compared to mammography alone), and massive data files with related storage and processing issues and delays. Early research indicates that DBT combined with traditional mammography can decrease recall rates (greater confidence in the nature of benign lesions and capacity to disassemble composite densities) and boost cancer-detection rates. Current advances involve 2D mammogram image synthesis from the DBT data collection, contrast-enhanced DBT, and CAD for use in DBT. Few studies used DBT for BrC classification [182,183,184].

Kim et al. [118] proposed a latent bilateral feature representation using a 3D multi-view deep CNN (DCNN). The proposed DCNN was intended to identify obscured or latent bilateral attribute representations of masses in self-taught learning. The experimental findings revealed that the suggested latent bilateral feature representation outperforms traditional hand-crafted feature representations. However, they used limited images and tried to overcome the overfitting problem by using data augmentation. Sakai et al. [185] proposed ML-based models and a multi-layer perceptron (MLP) model for classifying breast lesions on digital breast tomosynthesis images. The benign tumors had a lower correct identification rate than the malignant tumors. The fact that benign tumors have a lower right-identification rate than malignant tumors may be due to a smaller number of benign cases than malignant cases.

### 4.6. Infrared Thermal Imaging (ITI)

The theory of infrared thermography is to measure the radiation released by a surface in an attempt to decide its temperature. In 1982, the Food and Drug Administration (FDA) confirmed infrared imaging as an alternative imaging modality to mammography [186]. Thermography has been used as a supplementary screening tool in BrC diagnosis. Ionizing radiation, venous entry, or other harmful techniques are not used to create infrared images. Aside from these details, it is painless and does not make contact with the skin’s surface, bringing little discomfort to the patient. It is less expensive than conventional tests such as mammography, ultrasound, and MRI [106]. At present, mammography is the second most popular means of screening for BrC. However, because of the poor contrast caused by the thick breast, mammography is not recommended for young women, and alternative methods must be considered [187]. Thermography has benefits for diagnosis in young women since thick tissues make early X-ray visualization of symptoms difficult. For example, microcalcifications and masses are typically noticeable only in mammograms of women of non-reproductive ages. As a result, digital infrared thermal imaging (DITI) as an imaging modality has the potential to overcome the limitations of mammography [142].

Furthermore, thermography is very helpful for identifying non-palpable BrC, which other tests cannot identify. Infrared imaging in conjunction with a powerful computer-aided device (CAD) will result in a highly accurate tumor detector [158]. DMR-IR is a popular dataset with infrared images that many studies utilized in BrC classification. However, these studies are based on machine learning. Few studies adopted deep learning as their BrC classifier with thermal images [142,187]. Mambou et al. [158] utilized the DMR-IR dataset with an InceptionV3-KNN ensemble model and demonstrated that their model could easily identify a breast as sick or healthy. Ekici and Jawzal [187] applied CNN for thermographic BrC screening. According to their results, thermography is a viable substitute for conventional BrC detection techniques since temperature variations allow for better localization of cancer/tumor cells, despite the fact that such cells undergo angiogenesis. Mambou et al. [142] suggested a transfer learning-based CNN (FTM-LL) that obtained a powerful binary classification (sick breast or healthy breast) with thermal breast images. However, the dataset they utilized included images of people aged 29 to 85, omitting young women.

### 4.7. Computed Tomography (CT)

The primary purpose of CT in BrC staging is as a whole-body examination. CT body scans are utilized in the staging of primary BrC to determine metastatic spread, monitor treatment response, and evaluate probable recurrence. A CT scan is a type of X-ray that is performed using a large X-ray machine. CT scans are sometimes known as CAT scans. CT scans are more common than MRIs and are usually less costly. On the other hand, MRI is believed to be superior in terms of image detail [176]. Modern multidetector CT scanners can identify both visceral and bone metastases. Dedicated breast CT scanners have been created to produce three-dimensional images of the breasts at a radiation dosage comparable to conventional mammography. Early studies have yielded encouraging results. However, the machines are costly and have yet to be incorporated into thea National Health Service (NHS). A study by Cong et al. [188] created a ResNet network to restore images from a few-view breast CT. The suggested network model yielded impressive results and has significant value in clinical breast-imaging applications. De Buck et al. [189] proposed a novel approach that integrates artificial deep learning-based breast segmentation from CT thorax exams with radiodensity and volumetric breast density-based breast glandular computation. A cutting-edge CNN was trained to segment the breast area, allowing them to compute BrC risk scores on the segmented CT volume. They also demonstrated that the proposed approach would correctly forecast two risk measures that include predictions of the likelihood of having BrC later in life. Despite the small set of training data, segmentation outcomes in the test set were relatively decent, with an overall dice score of 0.84.

### 4.8. Mass Spectrometry Imaging (MSI)

Breast-cancer patients have a better prognosis if they are discovered early, before the disease progresses to a point where modern medicine can no longer help; there is a need to find a reliable breast cancer tissue target. Mass-spectrometry (MS) technologies can access plasma and tissue from patients for breast cancer diagnosis and prognosis. For breast cancer diagnosis, ambient mass spectrometry imaging (MSI) and liquid chromatography mass spectrometry (LC-MS) are reliable and repeatable methods [190]. A study by Al-Wajeeh et al. [191] proved that information of protein expression, particularly in breast cancer stages 2 and 3, can provide critical hints that may aid the discovery of novel biomarkers in carcinogenesis. However, this imaging method has not been utilized with deep learning for breast cancer diagnosis. A study by Behrmann et al. [192] proposed a model for tumor classification with MSI, which was compared to a standard deep-learning approach, and it displayed superior performance. Another study by Silva et al. [190] experimented with different MSI techniques. The authors showed desorption-electrospray ionization mass-spectrometry imaging (DESI-MSI) as a reliable method for diagnosing breast cancer, including the precise classification of unique-type carcinomas based on their cancer status with high accuracy, sensitivity, and specificity.

### 4.9. Multi-Modalities (MM)

Aside from using a single medical-imaging modality to characterize BrC, some researchers have preferred to use at least two separate imaging modalities. We referred to this as multi-modality. Early research suggests that DBT combined with traditional mammography can decrease recall rates while improving cancer-detection rates [193]. Hadad et al. [117] showed the potential of a network trained with mammography images to distinguish masses in breast MRI images. The limitation of this work is the comparatively small scale of the dataset, which did not allow for the evaluation of additional scenarios such as transferring learned models from MRI to mammography and from site-specific subsets of the data. An integrated BrC program is being developed in this work to aid in the diagnosis and identification of breast cancers [134]. Mammograms and ultrasound images are considered from the mini-MIAS and BCDR databases. Furthermore, a model trained with multiple datasets incorporating multiple modalities is highly resilient in classifying real-world photos. As a result, certain frameworks can be trusted to be applied in real life.

## 5. Evaluation Metrics and Result Analysis

Evaluating a model is a critical step in developing an efficient deep-learning model. Following image pre-processing, training, and validation, the test images are input into the trained model for classification to evaluate its performance. There are various evaluation metrics, such as the confusion matrix, cross-validation, the receiver operating characteristic curve (ROC), the area under the ROC curve (AUC), and so on. The following terms in confusion metrics are frequently used to calculate evaluation metrics: true negative (TN), i.e., the test cases were negative as well as the prediction of the classifier; true positive (TP), i.e., the test cases were positive as well as the prediction of the classifier; false negative (FN), i.e., the test cases were positive, but the prediction of the classifier was negative; and lastly, false positive (FP), i.e., the test cases were negative, but the prediction was positive. The accuracy, precision, recall, and F1-score derived from the confusion matrix are popularly utilized to assess the model of BrC classification. These metrics are briefly listed in the following paragraphs.

**Accuracy (A):** The accuracy score is determined by dividing the percentage of correct predictions by the model’s total amount of predictions. It simply displays the percentage of normal patients who are accurately predicted and the percentage of abnormal BrC patients who are correctly diagnosed. The accuracy can be defined as in Equation (1).

(1) $$Accuracy\left(A\right)=\frac{TruePositive+TrueNegative}{Total}$$

**Precision (Pr):** Precision is determined by dividing the true positive outcomes by the actual positives results, including those incorrectly identified by the classifier. Precision can be expressed using Equation (2).

(2) $$Precision\left(Pr\right)=\frac{TruePositive}{TruePositive+FalsePositive}$$

**Sensitivity (Sn) or Recall (R):** The recall is measured as the proportion of true-positive results to the actual positives samples that should have been detected. To minimize the misdiagnosis of malignant patients, both Sn and Pr should be high during medical image diagnosis. The recall can be computed using Equation (3).

(3) $$Recall\left(R\right)=\frac{TruePositive}{TruePositive+FalseNegative}$$

**F1-score:** The F1-score determines the model’s accuracy in each class. The F1-score metric is usually used when the dataset is imbalanced. It is useful to compare two models that have a high Sn but a low Pr. It can be defined by Equation (4).

(4) $$F1-score\left(F\right)=2\times \frac{precision\times recall}{precision+recall}$$

**ROC curve and AUC:** The receiver operating characteristics (ROC) curve is used to show the success of a classification model across several classification thresholds. The true-positive rate (Recall) and the false-positive rate (FPR) are used in this curve. AUC is an abbreviation for “area under the ROC curve.” In other words, the AUC tests the whole two-dimensional field under the entire ROC curve. The FPR can be defined as in Equation (5).

(5) $$FPR=\frac{FalsePositive}{FalsePositive+TrueNegative}$$

**Patient score:** Assuming $N_{P}$ is the total number of cancer images of patient P, if $N_{rec}$ cancer images are accurately identified for each patient, a patient score may be defined as in Equation (6).

(6) $$PatientScore=\frac{N_{rec}}{N_{P}}$$

**Patient-recognition rate:** The patient-recognition rate is defined by Equation (7) as the ratio of the sum of patient scores to the total number of patients.

(7) $$PatientRecognitionRate=\frac{\sum PatientScore}{TotalNumberofPatients}$$

**Image-recognition rate:** Let $N_{all}$ represent the number of cancer images in the test set. If the system successfully classifies $N_{rec}$ cancer pictures, the recognition rate at the image level can be expressed as in Equation (8).

(8) $$Image-recognitionrate=\frac{N_{rec}}{N_{all}}$$

**Decision-curve analysis:** Diagnostic and prognostic models are often assessed using accuracy measures that do not take into account clinical consequences. Decision-analytic techniques allow for the evaluation of clinical outcomes, but they frequently necessitate the collection of extra data and might be difficult to apply to models that produce a continuous output. Decision-curve analysis is an approach for evaluating and comparing prediction models that takes into account clinical implications, requires only the data set on which the models are evaluated, and can be applied to models with either continuous or dichotomous outcomes.

Decision-curve analysis assesses a prediction model for an event, usually by displaying a graphical plot of the net benefit versus the threshold probability. The minimum probability of an event at which a decision-maker would take a specific action is known as the threshold probability. The net benefit can be determined using Equation (9), as a weighted combination of true- and false-positives. Where $p_{t}$ is the threshold probability, and N is the total number of observations.

(9) $$NetBenefit=\frac{TruePositive-FalsePositive\times \frac{p_{t}}{1-p_{t}}}{N}$$

Further, Table 12 presents a surveyed summary of evaluation metrics and the performance of previous studies.

## 6. Challenges and Research Directions

This section outlines potential research directions that should be investigated to enhance the BrC classification results. Through deep excavation on this topic, the potential prospective study directions are pointed below.

- ANNs, autoencoders, DBNs, and CNNs are currently being used for BrC diagnosis. Deep-learning networks of other kinds, such as RNN, GANs, and clustering should be explored in this field.
- CNN is widely used in BrC classification due to its ability to extract useful features from images. We suggest that strategies based on various CNN architectures, as well as hyperparameter optimization, must be investigated.
- Model efficiency is greatly influenced by feature and classifier selection. Considering the selection of features and classifiers could also boost performance. A study suggested that the alternative-feature-reduction method linear discriminant analysis (LDA) may also be examined [152].
- Better segmentation models, such as U-net, have provided cutting-edge segmentation findings in a variety of computer vision datasets [196,197]. Furthermore, applying this technique involving various imaging modalities may boost BrC classification results.
- A large number of research works built CNNs from scratch (De-novo model), and some studies publicly shared their implementation [23,198]. Implementations should be freely accessible for reuse, helping researchers for future exploration.
- A study suggested that additional clinical data may boost the performance of the classifier [193]. Increasing data would improve model performance and help to experiment in core model performance more than in handling data scarcity.
- Same-domain transfer learning is a method of training that uses images resembling the target dataset. For example, it trains the model on X-ray images of various breast diseases, and then fine-tunes and trains it on breast-cancer X-ray images for BrC diagnosis. Same-domain transfer learning should be examined as it has achieved promising results recently [199].
- Datasets focused on mammography and histopathology are publicly available. In contrast, datasets based on other imaging modalities such as infrared thermal imaging, computed tomography, and digital breast tomosynthesis are not publicly available. Additionally, studies conducted using such imaging modularities go through unpublished datasets. The research datasets should be published for future knowledge exploration and implementation.
- Future studies may examine the potential of the thermography approach to diagnose cancer levels [186]. The thermography method is currently understood to focus on the metabolic processes of cancerous cells, which generate heat in the affected breast mass areas [200]. A study wants to extend their work by using a 0.5-sensitivity thermal camera to build a 3D structure of the breast to handle the issue of BrC classification [158]. Yet, there are also unanswered questions about what else the deep-learning application can do with thermograms.
- Another field of thermography that requires further investigation is the use of advanced dynamic infrared thermography (DIRT), which allows a non-invasive, painless measurement without the possibility of radiation. The use of different templates, such as CNN, mathematical texture elements, and Gabor coefficient classifiers, in conjunction with the DIRT, can be tested to see if they can detect breast cancer.
- 3D tomosynthesis, a newly emerging breast-imaging technology that creates a video by rebuilding images captured from various viewpoints, has proved to be much more effective than traditional mammograms in manual tumor detection in clinical practice. For BrC classification, mass spectrometry imaging is not employed with deep learning, although it did enable precise classification of distinct-type carcinomas. These imaging technologies need to be investigated in BrC classification.
- Multi-modality research must be investigated. Sutdies have also explored combining mammography and MRI imaging methods. Another intriguing expansion of this work will be to use mammography and MRI-based models as a foundation for analyzing tomosynthesis images, which are increasingly becoming a standard breast-imaging modality.

Finally, Table 13 presents employed architectural strengths and limitations in BrC diagnosis, as well as research directions in terms of the architectures.

## 7. Conclusions

Automatic the detection and classification of BrC remain a significant challenge for radiologists to diagnose accurately. In recent years, deep-learning-based techniques for BrC diagnosis have been developed to help radiologists. This review discussed neural networks (ANN, autoencoder, DBN, CNN, ELM, and GAN) used in BrC detection and classification. The most widely used CNN in BrC classification is divided into three categories (De-novo, TL, and RL) based on their training and learning procedures. Descriptions of used datasets and their pre-processing techniques were also presented in this review. The survey covered the recent studies related to breast cancer using deep learning and divided them, based on their used imaging modalities, into nine sections (Hp, Mg, Us, MRI, DBT, ITI, CT, MSI, and MM). The study described all imaging methods used in BrC classification, mentioning their pros and cons. The histopathology and mammography imaging modalities were further categorized based on the detection and classification of cancer cells. The study also gave insights on used evaluation metrics and model performance with a table. Finally, the study highlighted the current challenges and provided some research directions for further advancements in this domain. We postulate that with the introduction of newer deep-learning network designs, BrC categorization will remain an active study subject for a certain period. In what follows, deep-learning-based models have a far lower likelihood of producing inaccurate results. We strongly believe that this pondering review will aid researchers in the BrC classification and furnish toward achieving results.

## Figures and Tables

**Table 1:** A comparison of existing surveys based on breast cancer diagnosis.

Survey		Taxonomy	Datasets	Imaging Modalities	Evaluation Metrics	Challenges	Deep-Learning Architectures					
Ref.	Year						ANN	Autoencoder	DBN	CNN	ELM	GAN
[27]	2017	✗	✓	✗	✓	✗	✗	✗	✗	✗	✗	✗
[28]	2018	✗	✓	✗	✗	✓	✓	✓	✓	✓	✗	✗
[29]	2018	✗	✓	✓	✓	✗	✗	✗	✗	✗	✗	✗
[30]	2019	✗	✗	✓	✗	✓	✓	✗	✗	✓	✗	✗
[31]	2019	✗	✓	✓	✓	✓	✓	✓	✓	✓	✗	✗
[32]	2019	✗	✗	✗	✓	✗	✗	✓	✓	✓	✗	✓
[33]	2020	✗	✗	✗	✗	✓	✓	✗	✗	✓	✗	✗
[34]	2020	✗	✓	✓	✗	✓	✓	✗	✗	✓	✗	✗
[25]	2020	✗	✗	✗	✗	✓	✗	✓	✗	✓	✗	✗
[35]	2020	✗	✗	✗	✗	✗	✗	✗	✗	✓	✗	✗
[36]	2021	✗	✗	✗	✗	✗	✗	✗	✗	✓	✗	✗
[37]	2021	✗	✓	✓	✓	✓	✗	✗	✗	✓	✗	✗
[26]	2021	✗	✗	✗	✓	✗	✗	✓	✗	✓	✗	✓
Ours	-	✓	✓	✓	✓	✓	✓	✓	✓	✓	✓	✓

**Table 2:** State-of-the-art studies based on ANN architecture.

Reference	Dataset	Architecture	Category	Strength	Limitation
Abbass [40]	WBCD	MPANN	BrC diagnosis	Better generalization	Absence of feature engineering
Karabatak and Ince [41]	WBCD	AR+NN	BrC diagnosis	Reducing feature dimensions	Inadequate model evaluation
Rouhi et al. [44]	MIAS, DDSM	ANN	Mammography, image segmentation	Correctly identify small mass lesion	Insufficient images
Jafari-Marandi et al. [42]	WDBC	LS-SOED	BrC diagnosis	Driven to better decision-making	Inclusion of the dataset’s missing values
Becker et al. [43]	BCDR	ANN	Mammography, BrC detection	Correctly identify small mass lesion	Insufficient images

**Table 3:** State-of-the-art studies based on autoencoder architecture.

Reference	Dataset	Architecture	Category	Strength	Limitation
Xu et al. [45]	PD	SSAE + Softmax	Histopathology, nuclei patch classification, unsupervised learning	High-level feature learning	Limited images
Xu et al. [46]	PLOS 2018	SSAE + Softmax	Histopathology, nuclei detection, unsupervised Learning	Lower computation time	Imbalanced data
Cheng et al. [51]	PD	SDAE	Ultrasound, supervised learning, breast-lesion classification	Adequate model evaluation	Absence of model comparison
Kadam et al. [47]	WDBC	FE-SSAE-SM	Feature ensemble learning, BrC classification	Adequate evaluation	Absence of data-preprocessing techniques
Feng et al. [50]	BCC	SDAE + Softmax	Histopathology, nuclei classification, unsupervised feature learning	Utilizing robust features of breast cancer nuclei	Insufficient images

PD = private dataset, FE-SSAE-SM = feature ensemble learning based on stacked sparse autoencoders and softmax regression model.

**Table 4:** State-of-the-art studies based on DBN architecture.

Reference	Dataset	Architecture	Category	Strength	Limitation
Abdel-Zaher and Eldeib [54]	WBCD	DBN-NN	Unsupervised learning, supervised learning, BrC classification	Tested on several train-test partition	May suffer from overfitting issue
Zhang et al. [59]	PD	PGBM	Ultrasound (shear-wave elastography (SWE)), feature extraction, classifying breast tumor	Utilized a different ultrasound technique	Higher training time
Dhungel et al. [56]	DDSM-BCRP, INbreast	DBN	Mammography, segmentation of masses, structured learning	Can learn complex features	Inadequate model evaluation
Dhungel et al. [57]	DDSM-BCRP, INbreast	CRF	Mammography, segmentation of masses, structured output learning	Significantly faster model	Inadequate model evaluation
Al-antari et al. [58]	DDSM	DBN	Mammography, automatic mass detection	Feature engineering	Higher error rate for confusing benign with malignant
Khademi and Nedialkov [55]	WDBC, WOBC	DBN	Breast cancer diagnosis	The integration of microarray and clinical data	Comparison with ML models instead of other DL models

**Table 5:** State-of-the-art studies based on De-novo CNN.

Reference	Dataset	Architecture	Category	Strength	Limitation
Arevalo et al. [7]	BCDR	CNN (UDM)	Mammography, mass lesion classification	omparison with a pre-trained model	Simple architecture
Spanhol et al. [60]	BreakHis	CNN (UDM)	Histopathology, image classification	Used high-resolution histopathological images	For training, only small patches of the images are used.
Albarqouni et al. [72]	Crowdsourcing	AggNet	Histopathology, mitosis Detection	Tested with a benchmark dataset	unreliable (crowd) annotations
Xu et al. [65]	PD	DCNN (COM)	Histopathology, image segmentation and classification	Can learn complex features	Insufficient images
Kooi et al. [66]	PD	CNN	Mammography, breast mass lesion classification	Focused on the detection of solid, malignant lesions including architectural distortions	Absence of benign lesions in training set
Araújo et al. [71]	BICBH	CNN (UDM)	Histopathology, image-wise classification, patch-wise classification	Multi-class classification	Limited images
Samala et al. [67]	PD	DLCNN (UDM)	Digital breast tomosynthesis, recognition of microcalcification	Learns complex patterns	Limited images
Ting et al. [70]	MIAS	CNNI-BCC (UDM)	Mammography, breast-lesion classification	Feature-wise data augmentation	Limited images
Yan et al. [68]	PD	CNN+RNN	Histopathology, pathological image classification	Released a larger and more diverse dataset	Lack of data pre-processing
Wang et al. [69]	BreakHis	CNN (UDM)	Histopathology, BrC binary classification, deep feature fusion, and enhanced routing	Classification is conducted for different magnification factors	Absence of image pre-processing

**Table 6:** State-of-the-art studies based on a TL-based CNN.

Reference	Dataset	Architecture	Category	Strength	Limitation
Huynh et al. [63]	PD	AlexNet	Mammography, feature extraction, breast-mass classification	Automatic lesion segmentation	Inadequate model evaluation
Samala et al. [73]	DDSM	CNN (FTM-ML)	Mammography, mass classification	Multi-task transfer learning	Absence of model comparison
Chougrad et al. [77]	DDSM, BCDR, INbreast	VGG16, ResNet50 and Inception v3 (FTM-ML)	Mammography, mass-lesion classification	Merged three datasets	Inadequate model evaluation
Xie et al. [22]	BreakHis	CNN (FTM-LL)	Histopatology, multi-class classififaction, clustering analysis	Solved the unbalanced distribution of samples	Lack of image pre-processing
Mendel et al. [74]	PD	CNN (FTM-ML)	Mammography, digital breast tomosynthesis, classification	Leave-one-out step-wise feature selection was used to eliminate redundant features.	Lack of training data
Kumar et al. [78]	BreakHis	VGGNet-16 (FTM-ML)	Histopathology, feature extraction, image classification	Analysis of effects of image pre-processing	Accuracy is influenced by magnification
Yu et al. [64]	PD	CNN (FTM-ML)	Histopathology, image classification	Images are collected via the internet.	The quality of the images could be inadequate.
Hu et al. [61]	PD	CNN (FTM-ML)	MRI, feature extraction	Pre-processing, large dataset, and extended training times are not required	Issue of class imbalance

**Table 7:** State-of-the-art studies based on RL-based CNN.

Reference	Dataset	Architecture	Category	Strength	Limitation
Toğaçar et al. [79]	BreakHis	BreastNet	Histopathology, BrC diagnosis	Can be used in all microscopic images at different magnification rates	Absence of data pre-processing
Gour et al. [80]	BreakHis	ResHist	Histopathology, lassification of benign and malignant	Preserves the global information of histopathological images	Consumes a lot of processing power
Hu et al. [81]	BreakHis	myResNet-34	Histopathology, malignancy-and-benign classification	Automatic target image generation	Significant rate of misclassification
Singh et al. [83]	PD	ResNet	Mammography, digital breast tomosynthesis, multi-class classification	The approach is simple and can be applied in different imaging	Only patch-level images are used to train the model.
Li et al. [84]	PD, INbreast	ResNet50	Mammographic density classification	Combination of deep residual networks with integrated dilated convolutions and attention methods	Imbalance classes

**Table 8:** State-of-the-art studies based on ELM architecture.

Reference	Dataset	Architecture	Category	Strength	Limitation
Lahoura et al. [85]	WBCD	ELM	Feature selection, cloud environment, BrC diagnosis	Consideration of feature engineering	Absence of image pre-processing technique
Wang et al. [89]	PD (Mamograms)	ELM	Mass detection, feature extraction, Clustering	Feature fusion	Insufficient data
NEMISSI et al. [87]	WBCD	ELM	BrC diagnosis, genetic algorithm	Higher generalization performance	Inadequate evaluation
Ronoud and Asadi [90]	WDBC	ELM (DBN+ELM+BP)	BrC diagnosis, ensemble approach	Parameter tuning	
Wang et al. [88]	BreaKHis, ImageNet	ELM (ICELM)	Feature extraction, double-step deep transfer learning, BrC diagnosis	A novel method	Not an end-to-end architecture
Toprak [86]	WBCD	ELM	Detection and characterization of benign and malignant types	ELM is superior to other methods in performance and speed	Imbalance classes
Muduli et al. [91]	WBCD	ELM	Classification of breast masses, feature extraction and reduction	The generalization performance is improved	There is a possibility of data loss

**Table 9:** State-of-the-art studies based on GAN architecture.

Reference	Dataset	Architecture	Category	Strength	Limitation
Guan and Loew [96]	DDSM	GAN	Image augmentation, BrC diagnosis	Sufficient images	GAN is only used as image generator
Shams et al. [93]	WBCD	GAN (DiaGRAM)	BrC diagnosis	Enhanced feature learning	
Thuy and Hoang [95]	BreaKHis	GAN (styleGAN, Pix2Pix)	Image augmentation	Feature extraction with VGG16 and VGG19	Generated images contain noise and affected the classifiers accuracy
Singh et al. [94]	DDSM, INbreast	GAN (cGAN)	Breast-tumor segmentation	Works well on limited training samples	Tumor segmentation from full-mammograms has a low accuracy
Fan et al. [97]	PD (DCE-MRI images)	GAN (SRGAN)	Image augmentation, BrC diagnosis	Generated super resolution ADC images	There is no conventional medical process that uses ADC images
Swiecicki et al. [98]	PD	GAN	Digital breast tomosynthesis, image completion, abnormality detection	Able to identify suspicious regions without the need for training images containing abnormalities	Inadequate model evaluation
Tien et al. [99]	PD	GAN	Computed tomography image-quality improvement	Can convert blurred images into clear images	Only for chest region

PD (DCE-MRI) = private dataset of dynamic contrast-enhanced magnetic resonance imaging, ADC = apparent diffusion coefficient.

**Table 10:** Detailed information of publicly available datasets.

SL	Dataset Name	Category	No. of Images	Classes	Image Format	Resolution	URL
1	DDSM [107]	Mammograms	10,480	Benign, cancer, normal, benign without callback (bwc)	.JPEG	16-bit	http://www.eng.usf.edu/cvprg/Mammography/Database.html (accessed on 1 October 2021)
2	MIAS [102]	Mammograms	322	Benign, malignant, normal	.PGM	8 bit	https://www.repository.cam.ac.uk/handle/1810/250394 (accessed on 1 October 2021)
3	mini-MIAS [103]	Mammograms	322	Calcification, circumscribed masses, spiculated masses, other/ill-defined masses, architectural distortion, asymmetry, normal	.PGM	1024 × 1024 pixels	http://peipa.essex.ac.uk/info/mias.html (accessed on 1 October 2021)
4	CBIS-DDSM [101]	Mammograms	1644	Normal, benign, and malignant	.DICOM	16-bit	https://wiki.cancerimagingarchive.net/display/Public/CBIS-DDSM (accessed on 1 October 2021)
5	INBreast [108]	Mammograms	410	Benign, malignant, normal	.DICOM	14-bit	http://medicalresearch.inescporto.pt/breastresearch/index.php/Get_INbreast_Database (accessed on 1 October 2021)
6	UPMC	Tomography and mamograms	- -	Hamartoma, invasive ductal carcinoma (IDC), asymmetry, lobular carcinoma, papilloma, calcifications	.DICOM	-	https://www.dclunie.com/pixelmedimagearchive/upmcdigitalmammotomocollection/index.html (accessed on 7 October 2021)
7	BICBH [71]	Histology images	259	normal, benign, in situ carconima and invasive carcinoma	.TIFF	- -	https://rdm.inesctec.pt/dataset/nis-2017-003 (accessed on 7 October 2021)
							http://www.bioimaging2015.ineb.up.pt/dataset.html (accessed on 7 October 2021)
8	BreakHis [109]	Histology images	7909	Benign and malignant	.PNG	8-bit	https://web.inf.ufpr.br/vri/databases/breast-cancer-histopathological-database-breakhis/ (accessed on 10 October 2021)
9	BCC [110]	Histology images	58	Malignant, benignant	.TIFF	896 × 768 pixels, 768 × 512 pixels	http://bioimage.ucsb.edu/research/bio-segmentation (accessed on 10 October 2021)
10	BACH [111]	Histology images	400	Normal, benign, in situ carcinoma, invasive carcinoma	. TIFF	2048 × 1536 pixels	https://iciar2018-challenge.grand-challenge.org/Dataset/ (accessed on 10 October 2021)
11	TUPAC16 [112]	Histology images	500	- -	.SVS	50 k × 50 k pixels	https://tupac.grand-challenge.org/Dataset/ (accessed on 10 October 2021)
12	IDC [14]	Histology images	162	Invasive ductal carcinoma (IDC), non-IDC	.PNG	- -	https://www.kaggle.com/paultimothymooney/breast-histopathology-images (accessed on 14 October 2021)
13	MITOS-ATYPIA 14	Histology images	-	Mitosis and nuclear atypia	.TIFF	1539 × 1376 pixels, 1663 × 1485 pixels	https://mitos-atypia-14.grand-challenge.org/dataset/ (accessed on 14 October 2021)
14	DMR-IR	Infrared Images	- -	- -	- -	640 × 480 pixels	http://visual.ic.uff.br/en/proeng/thiagoelias/ (accessed on 15 October 2021)
15	BCDR [105]	Mammograms and ultrasound	- -	Benign, malignant, normal	.DICOM	720 × 1167	https://bcdr.ceta-ciemat.es/information/about (accessed on 15 October 2021)
16	TCGA	Mammograms	88	- -	.DICOM	- -	http://cancergenome.nih.gov/ (accessed on 24 October 2021)
17	BancoWeb LAPIMO [113]	Mammograms	1473	Benign, malignant, normal	.TIFF	12 bits	http://lapimo.sel.eesc.usp.br/bancoweb/ (accessed on 24 October 2021)
18	PLOS 2018	Histology images	537	Nuclear, non-nuclear	.TIFF	2200 × 2200 pixels	https://engineering.case.edu/centers/ccipd/data (accessed on 27 October 2021)
19	WBCD or WBCO [100]	Multivariate	699 data	Benign, malignant	- -	- -	https://archive.ics.uci.edu/ml/datasets/breast+cancer+wisconsin+(original) (accessed on 3 October 2021)
20	WDBC [114]	Multivariate	569	Malignant, benign	- -	- -	https://archive.ics.uci.edu/ml/datasets/Breast+Cancer+Wisconsin+(Diagnostic)/ (accessed on 3 October 2021)
21	Histopathological images [68]	Histology images	3771	Normal, benign, in situ carcinoma and invasive carcinoma	- -	2048 × 1536 pixels	http://ear.ict.ac.cn/?page_id=1616 (accessed on 3 October 2021)

**Table 11:** The advantages of image pre-processing methods used in previous studies are presented.

Pre-Processing Method	Methodology	Advantages	References
Image augmentation	Geometric transformations such as rotation and flipping	To prevent the problem of overfitting. To address the issue of class imbalance in training. For improved interpretation of HP images, the network can learn lesions from several perspectives, much like a pathologist does in real life.	[7,23,50,65,68,70,71,73,78,80,115,116,117,118,119,120,121,122,123,124,125,126,127,128,129,130,131]
	Insert noise/distortion (Gaussian noise, barrel or pin cushion transforms)	Allows for the robust training of NN It can predict with greater accuracy even when images are noisy	
	Patch-creation methods (patches with 50% overlapping, no overlapping, or randomly selected patches)	It can retain the image aspect ratio, architecture, or shape of the lesion, as well as subjective information. It improves the classifier’s performance while decreasing the likelihood of false negatives. It can decrease the possibility of information loss.	
	Synthetic minority over-sampling technique (SMOTE)	To solve the class imbalance problem before training NNs, this method increases the number of samples to the minority class.	
ROI extraction	Methods such as region growing, nuclei segmentation, the Otsu method, and the Markov random model were utilized.	Increases the amount of positive and negative image samples available. Assists the neural network (NN) in learning better representations of abnormal areas and decreases the likelihood of overfitting. Reduces calculation time and resource use.	[7,44,50,51,70,73,116,118,119,120,121,124,127,132,133,134,135,136,137,138,139,140,141,142,143]
Scaling	Gaussian pyramid, bi-cubic interpolation, bilinear interpolation	The image must be resized before being provided as input to the NN. Carefully chosen interpolation algorithms can prevent information loss while mapping to the new pixel grid. Along with resizing, the Gaussian pyramid can assist to increase the number of images.	[51,59,65,118,119,120,121,122,125,126,133,135,136,144,125]
Normalization and enhancement	Histogram equalization, adaptive mean, median filters, log transforms, CLAHE method, Wiener filter, multi-threshold peripheral equalization algorithm.	Normalize the image’s low-value and high-value intensity/contrast. Adaptive filters reduce noise by taking into account mean, variation, and spatial correlations. Reduces the effects of image blurring and impulsive noise in ultrasound images. Multi-threshold peripheral equalization enhances and removes irrelevant information from mammograms. On the normalized image, ANN typically performs better. It aids in the reduction in loss during backpropagation.	[7,44,58,62,89,115,120,122,125,131,133,134,139,143,145,146,147,148,149,150,151,152]
Remove artifacts	Using binary images and thresholding the pixel intensity, cropping border, extracting larger regions, using geometric parabola around rib cage.	Non-breast areas (labels, wages, white strips/borders, opaque markers, lungs, thorax, chest wall, and pectoral muscle) in mammograms, US, and MRI can be reduced.	[51,121,127,133,153]
Stain normalization or removal	Stain normalization	To make variable color (due to H&E staining of histology images) uniform across all images for certain patients. As a consequence, NN will not be distracted by variations in brightness and color staining and will produce superior classification results for multiclass BrC. The contrast, intensity, and color characteristics of the source images are almost identical to those of the reference image.	[78,115,116,121,129,130,154]
	Color deconvolution	To extract hematoxylin-eosin (H&E) staining intensities from HP images and to transform them into optical density space images without being considerably affected. It decreases image dimensionality, consumes fewer resources, and improves classification performance. It maintains textural information in histology images that is related with stain colors.	

**Table 12:** An overview of datasets, references, methods, evaluation metrics, and accuracy.

Dataset	Reference	Evaluation Metrics	Methods	Accuracy
MIAS, DDSM	Rouhi et al. [44]	Sensitivity, specificity, accuracy, AUC	ANN	90.16%, 96.47%
DDSM	Kumar et al. [119]	Accuracy	ANN	100%
BCC	Feng et al. [50]	Precision, recall, F1 Score, accuracy, mean execution time	autoencoder	98.27%
-	Wu et al. [194]	Accuracy, sensitivity, specificity, and autoencoder	95.45%	
WBCO, WDBC	Ronoud and Asadi [90]	Accuracy, sensitivity, specificity, NPV, PPV,	DBN+ELM	99.75%
DDSM	Mandala and Di [195]	Sensitivity, specifity, accuracy	DBN	93%
DDSM, PD (DBT)	Samala et al. [67]	ROC, AUC	CNN (UDM)	0.93 (AUC)
BreakHis	Spanhol et al. [60]	Accuracy, patient score, patient-recognition rate, image-recognition rate	CNN(UDM)	90%
MIAS	Ting et al. [70]	Accuracy, sensitivity, specificity	CNN (UDM)	90.50%
PD	Yan et al. [68]	Accuracy, sensitivity, AUC	CNN (UDM)	91.3%
BreakHis	Han et al. [126]	Recognition rates, accuracy	CNN(COM)	96.9%
BreakHis	Kumar et al. [78]	Accuracy, F1-score	CNN (TL)	97.01%
BreakHis	Toğaçar et al. [79]	Accuracy, sensitivity, specificity, precision, and F1-score	CNN (RL)	98.70%
BreaKHis	Hu et al. [81]	Precision, recall, accuracy, F1-Score	CNN (RL)	91%
BreaKHis	Wang et al. [88]	Accuracy, sensitivity	ELM	98.18%
WBCD	Lahoura et al. [85]	Accuracy, recall, precision, F1-score	ELM	98.68%
DDSM, INbreast	Shams et al. [93]	Accuracy, AUC	GAN+CNN	89%, 93.5%
DDSM, INbreast	Singh et al. [94]	Accuracy	cGAN	80%

**Table 13:** A research directive for architectural selection for BrC diagnosis.

Architecture/Policy	Architecture Strength	Architecture Limitation	Research Direction
ANN	Driven to better decision-making.	Not suitable for extracting spatial information	Architectures suitable for extracting spatial information are required.
Autoencoder	Excellent for condensing feature information.	Requires separate feature classification system, along with fine-tuned multi-stage training strategies.	Implementation of a single-stage training platform is required.
DBN	Requires low data on training.	DBN is not incredibly optimized for image-recognition processes.	Requires stronger fusion with convolutional architectures.
Transfer learning	Strong weight initialization results in achieving better accuracy from minimal training data.	Requires intuition of feature relation between pre-trained dataset and target dataset.	Transfer learning strategies should be implemented based on data relevancy.
Residual learning	Enables generalization of deeper architectures by auto-calibration on unnecessary features.	Requires batch-normalization resulting in adding extra computation complexity.	Unnecessary and heavy residuals should be avoided.
ELM	Faster learning capability with the advantage of avoiding vanishing gradients.	Hard to solve underfitting and overfitting issues. Additionally, ELM is not great at image-classification tasks.	Need to move towards deep-learning strategies or extensively improve architectural perspectives.
GAN	Excellent for data distribution learning and generating synthetic data.	Training sometimes falls into local-minima. Additionally, it may cause excessive focus on fixed data patterns.	Should be implemented for generating synthetic datasets and increasing the capability of cancer classifier models.

## Data Availability

There is no statement regarding the data.
